# Global spatial dataset of mangrove genus distribution in seaward and riverine margins

**DOI:** 10.1038/s41597-024-03134-1

**Published:** 2024-03-20

**Authors:** Alice Twomey, Catherine Lovelock

**Affiliations:** School of the Environment, The University of Queensland, Brisbane, Queensland 4067 Australia

**Keywords:** Ecosystem ecology, Environmental sciences

## Abstract

Mangroves are nature-based solutions for coastal protection however their ability to attenuate waves and stabilise and accrete sediment varies with their species-specific architecture and frontal area. Hydrodynamic models are typically used to predict and assess the protection afforded by mangroves, but without species or genus distribution information, the results can be significantly different from reality. Data on the frontal genus of mangroves exposed to waves and tides can provide information that can be used in hydrodynamic models to more accurately forecast the protection benefit provided by mangroves. Globally, frontal species were identified from existing mangrove zonation diagrams to create a global mangrove genus distribution map. This dataset aims to improve the accuracy of hydrodynamic models. Data may be of interest to researchers in coastal engineering, marine science, wetland ecology and blue carbon.

## Background & Summary

Globally, cities are looking to adopt nature-based solutions for coastal protection^1^, mitigating the increasing erosion and flooding caused by climate change^2^. Mangroves have received much attention, but their ability to attenuate waves and reduce flooding is highly variable based on their areal extent, water depth and stage of the tide^3^, density^4^, and genus-specific architecture^5^. Species in genera with large aboveground root systems, and therefore frontal areas, such as *Rhizophora* spp. have reported attenuating nearshore waves heights by up to 70%^6^, whereas species in genera with a much smaller frontal area such as *Avicennia* spp., typically have a reduced effect on waves comparatively^7^. To identify the viability of mangrove forests as nature-based solutions, hydrodynamic models used to predict erosion and flooding need to account for mangrove tree architecture variability based on characteristics of genera and species. However, there is currently no global map of the distribution of mangrove genera/species that occupy the seaward margins of mangroves.

Hydrodynamic models are used to predict the effect of vegetation on flooding and erosion of coastlines but typically represent vegetation such as mangroves as a ‘drag coefficient’^8^ or as ‘2D rigid cylinders’^9–11^. While both of these methods are state of the art, they are not accurate unless they are specific to genera/species of mangrove which are exposed to the waves, termed the ‘frontal species’^12^. The ability of mangroves to withstand coastal hazards is primarily driven by the influence of the architecture of the frontal vegetation within a forest. While there is much data identifying the architecture of specific mangrove genera and species^13,14^ and datasets showing mangrove distribution^15^ or biomass and canopy height^16^, there is currently no spatial dataset highlighting the distribution of mangrove genera/species.

Mangrove species typically occur in a discrete order (seaward to landward) based on intertidal environmental factors, including hydroperiod, salinity, soil-type, sedimentation, nutrient availability, propagule predation^17–20^ and propagule dispersal^21,22^. Geomorphologists have typically created mangrove zonation diagrams that illustrate the position within the intertidal zone or ‘order’ in which the mangrove species/genera exist, which is known to vary within and among regions^23^. Although these diagrams are qualitative, they provide an important as yet unutilised resource. This dataset therefore brings together information from zonation patterns described globally to create a single spatial layer of mangrove frontal species. This dataset provides a global spatial layer identifying the frontal mangrove species for each marine ecoregion of the world (MEOW)^24^. The outputs include a spatial layer of frontal mangrove genera, and species where available, a comprehensive dataset of all mangrove zonation data and an interactive spatial model illustrating the location of zonation diagrams using ArcGIS StoryMaps.

The benefits of this spatial layer are two-fold; 1) a new global spatial layer to include the species of mangroves, and 2) this can be used in coastal engineering models to more accurately prescribe the roughness factor, drag or architecture in coastal engineering models. This spatial layer has value for both marine ecologists, coastal engineers and conservation scientists.

## Methods

A systematic literature search was conducted to identify published diagrams outlining the mangrove species zonation observed for a given area. The data extracted from these diagrams included the location, mangrove species present, discrete order of the mangrove species within the intertidal zone (from their seaward to landward location) and marine ecoregion^24^. ArcMap 10.8^25^ software was used to develop the Bunting, *et al*.^15^ mangrove presence spatial data into a mangrove species-specific map, categorised by marine ecoregions^24^.

### Structured literature search

A systematic literature search was conducted using the University of Queensland’s library search tool, which queries databases such as Web of Science and SCOPUS. The search was conducted on and before 1^st^ November 2022.

### Search criteria

Topic search criteria included the following terms: (‘country name’) AND (‘mangrove + zonation’ OR ‘mangrove + profile’). All countries that have an ocean border (141 total) were included in the ‘country name’ search criteria. If any combination of two or more search terms appeared in the title or abstract, the article was shortlisted and later read in total to identify eligibility.

### Eligibility for inclusion

Articles returned by the literature search were included in the meta-analysis if they included a diagram or image of a mangrove profile illustrating the zonation of different mangrove species. The review yielded 195 eligible studies and 510 zonation diagrams.

### Attaching locations to each zonation observation

Specific locations of the zonation observation were recorded for 68 of the diagrams. For most of the zonation diagrams, the exact latitude and longitude were not recorded but included the location name. Google Earth was used to visually inspect the area outlined in the article and a location where mangroves appeared to be present was selected. Observations whose locations have been selected in this manner have been highlighted in the original Excel dataset.

### Relevance for inclusion

Google Earth was used to visually inspect each the location of each mangrove transect to ensure mangroves were present in the area and relevant. For instances where a mangrove zonation diagram was relevant to a region larger than several MEOW such as for ‘climatic regions’, these data were omitted from the spatial layer but still included in the original Excel dataset. These observations have been highlighted by including a description of where they are relevant but with ‘NA’ in the latitude and longitude.

### Developing the spatial layer

Using ArcMap 10.8, the marine ecoregion^24^ spatial layer was overlayed onto the mangrove presence spatial layer^15^. The marine ecoregions mapped by Spalding, *et al*.^24^ were used as a proxy for the varying conditions which may contribute to mangrove zonation. Marine ecoregions where mangroves from Bunting, *et al*.^15^ were not present, were removed from the dataset.

**Fig. 1:**
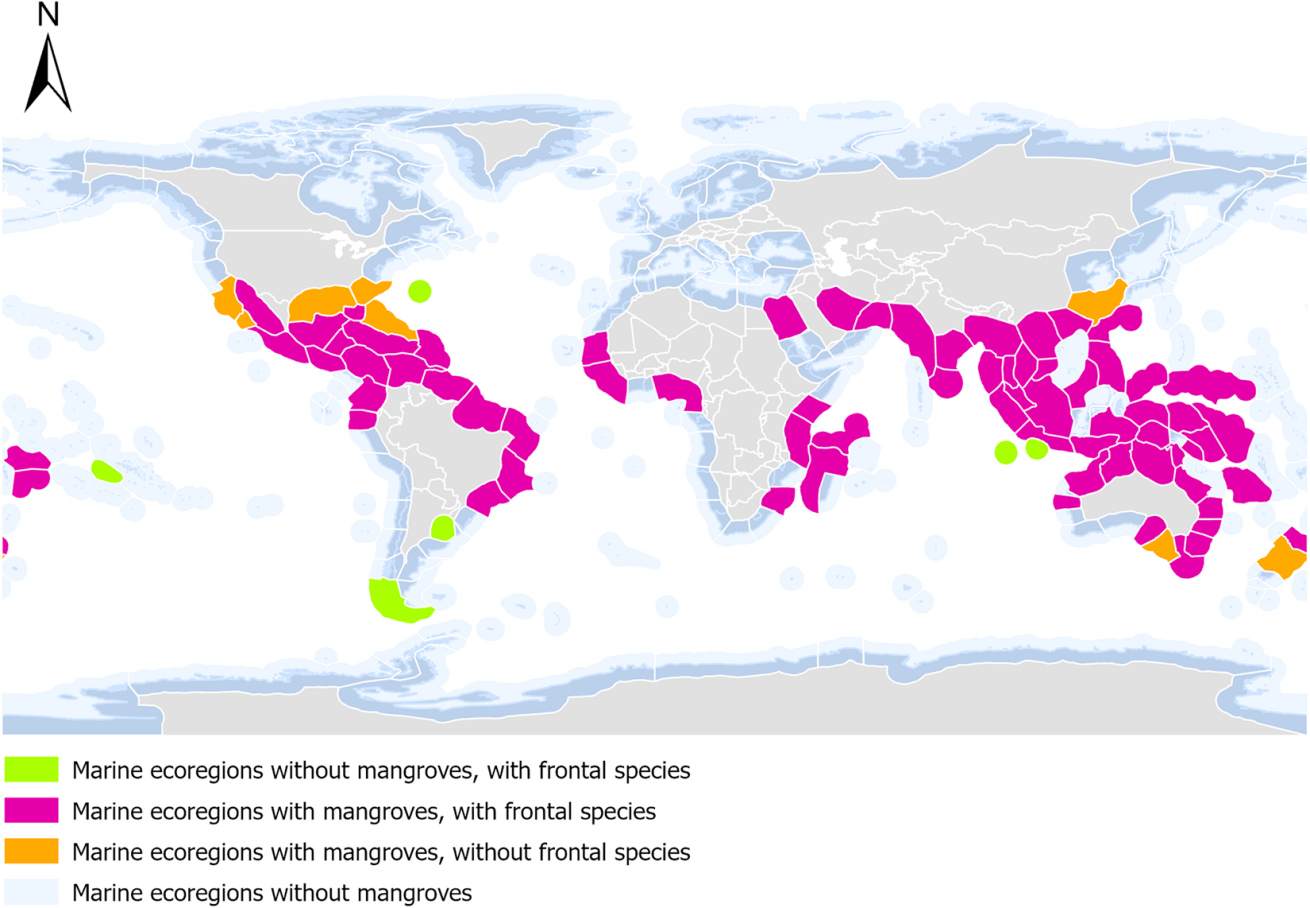
Availability of mangrove species zonation data for each marine ecoregion of the world.

Using the latitude and longitude of each mangrove zonation observation, a frontal species was assigned to the relevant marine ecoregion (Fig. 1). Mangrove frontal species were selected based on the diagram from the relevant marine ecoregion. Where multiple diagrams exist for the same marine ecoregion generally showing similar zonation patterns, the most common species was adopted. Where numerous diagrams existed for the same marine ecoregion, but there were few similarities amongst genus zonation patterns reported, the marine ecoregion was divided into separate polygons to accommodate the site-specific frontal genera. For these areas, the MEOW polygon was split equidistant from each key record of frontal species. For these areas, the MEOW polygon was split equidistant from each key frontal species. Where no data exists for a given ecoregion, no mangrove species was attached.

## Data Records

**Fig. 2:**
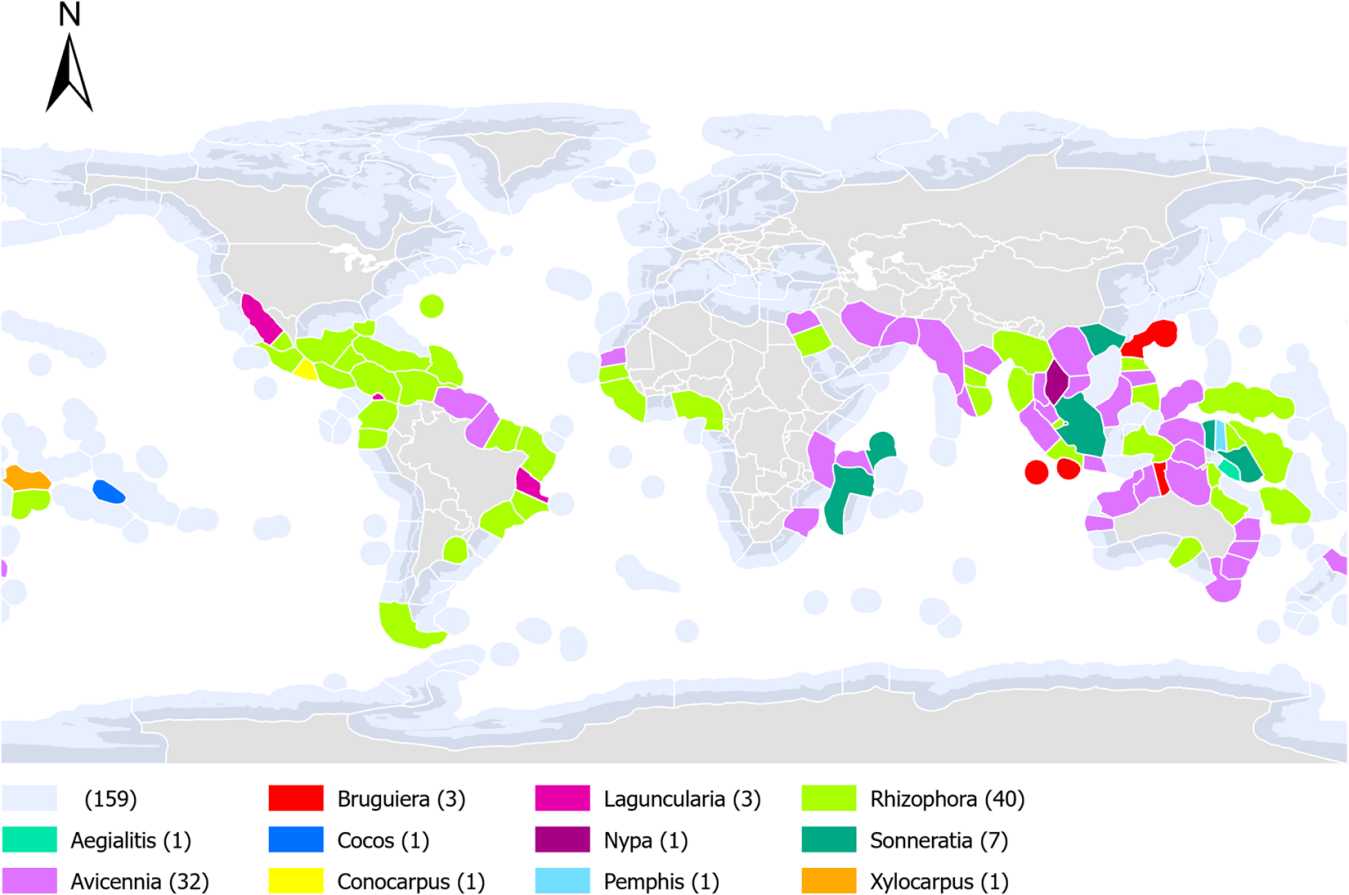
The distribution of the dominant mangrove genera that form seaward fringing (frontal stands) for each marine ecoregion of the world. The light blue shading (159) represents the Marine Ecoregions of the World where studies of mangrove zonation were not found. Values in parentheses indicate the number of regions for that genus.

The frontal mangrove genus spatial dataset^26^ is available at the PANGAEA online data repository in the form of a shapefile: FrontalMangroveSpecies.shp (Fig. 2). Each record (each line in the associated attribute table) corresponds to a marine ecoregion with three columns, ‘Genus_1’, ‘Genus_2’ and ‘Genus_3’ showing the dominant frontal genus. In several instances, multiple mangrove zonation diagrams existed within the same marine ecoregion, so the most common frontal mangrove species was adopted. Where several species occurred within the same region, if there was an obvious spatial divide, the marine ecoregion was split. Where there were multiple species of approximately equal numbers in the same ecoregion occurring at approximately the same location, each dominant genus was listed in a column in no particular order.

The original data derived from the mangrove zonation diagrams are available in an Excel file: MangroveZonationData.xlsx. Each record (each line in the file) corresponds to a mangrove zonation diagram published in the literature. The total number of records in the file is 510 across 195 articles.

An ArcGIS Story Map has been developed allowing the reader to view the original mangrove zonation diagrams with reference to their spatial relevance^27^.

## Technical Validation

The coordinates of all published observations of mangrove zonation included in this dataset were validated using Google Earth to confirm the presence of mangroves at the respective locations.

## Usage Notes

The shapefile format of the provided dataset enables linking the dataset to other spatial datasets, including shapefiles and rasters. The Excel format of the provided dataset enables data reproducibility and future updating of the shapefile.

## Data Availability

No custom code was used to generate or process the data described in the manuscript.
